# The structure stability of negative symptoms: longitudinal network analysis of the Brief Negative Symptom Scale in people with schizophrenia

**DOI:** 10.1192/bjo.2023.541

**Published:** 2023-09-07

**Authors:** Paola Rucci, Edoardo Caporusso, Francesco Sanmarchi, Giulia M. Giordano, Armida Mucci, Luigi Giuliani, Pasquale Pezzella, Andrea Perrottelli, Paola Bucci, Paola Rocca, Alessandro Rossi, Alessandro Bertolino, Silvana Galderisi, Mario Maj, Italian Network for Research on Psychoses

**Affiliations:** Department of Biomedical and Neuromotor Sciences, University of Bologna, Bologna, Italy; Department of Psychiatry, University of Campania ‘Luigi Vanvitelli’, Naples, Italy; Department of Neuroscience, Section of Psychiatry, University of Turin, Turin, Italy; Department of Biotechnological and Applied Clinical Sciences, Section of Psychiatry, University of L'Aquila, L'Aquila, Italy; Department of Basic Medical Science, Neuroscience and Sense Organs, University of Bari ‘Aldo Moro’, Bari, Italy; Department of Psychiatry, University of Campania ‘Luigi Vanvitelli’, Naples, Italy

**Keywords:** Schizophrenia, negative symptoms, Brief Negative Symptom Scale, network analysis, structure stability

## Abstract

**Background:**

The structure of negative symptoms of schizophrenia is still a matter of controversy. Although a two-dimensional model (comprising the expressive deficit dimension and the motivation and pleasure dimension) has gained a large consensus, it has been questioned by recent investigations.

**Aims:**

To investigate the latent structure of negative symptoms and its stability over time in people with schizophrenia using network analysis.

**Method:**

Negative symptoms were assessed in 612 people with schizophrenia using the Brief Negative Symptom Scale (BNSS) at baseline and at 4-year follow-up. A network invariance analysis was conducted to investigate changes in the network structure and strength of connections between the two time points.

**Results:**

The network analysis carried out at baseline and follow-up, supported by community detection analysis, indicated that the BNSS's items aggregate to form four or five distinct domains (avolition/asociality, anhedonia, blunted affect and alogia). The network invariance test indicated that the network structure remained unchanged over time (network invariance test score 0.13; *P* = 0.169), although its overall strength decreased (6.28 at baseline, 5.79 at follow-up; global strength invariance test score 0.48; *P* = 0.016).

**Conclusions:**

The results lend support to a four- or five-factor model of negative symptoms and indicate overall stability over time. These data have implications for the study of pathophysiological mechanisms and the development of targeted treatments for negative symptoms.

Negative symptoms are a fundamental aspect of schizophrenia, closely linked to poor quality of life and ineffective response to treatment.^1–12^ Despite their significance, they are still an unmet need in schizophrenia care, burdening patients, families and healthcare systems.^2,13–17^ Negative symptoms can be either primary or secondary manifestations of the disease; in the latter scenario, they can be subsequent to positive and depressive symptoms, or extrapyramidal side effects.^2,6^ The conceptualisation of negative symptoms proposed in the early 1900s included two aspects: the reduction of emotional expression and the loss of motivation.^18^ Indeed, Eugen Bleuler reported that people with schizophrenia had expressionless faces, were apathetic and lacked the desire to act on their own initiative or at the request of another.^19^ Emil Kraepelin described the presence of emotional apathy and a decline in volitional control in the same population.^20^ Contemporary understanding, stemming from the Measurement and Treatment Research to Improve Cognition in Schizophrenia (MATRICS) initiative developed by the National Institute of Mental Health (NIMH), posits five domains: blunted affect, alogia, avolition, asociality and anhedonia.^21^ Second-generation rating scales, such as the Brief Negative Symptom Scale (BNSS)^22^ and the Clinical Assessment Interview for Negative Symptoms (CAINS),^23^ were developed according to the NIMH-MATRICS consensus statement^21^ to provide an accurate assessment of negative symptoms in their quantitative (frequency, duration and intensity) and qualitative aspects (such as differentiation between anticipatory and consummatory aspects of anhedonia or differentiation between behavioural and experiential aspects).^22,23^

## The evidence base for the two- and five-factor models

The negative symptom structure has been widely investigated and exploratory factor analytic studies supported a two-factor model comprising a motivation and pleasure dimension (MAP, including avolition, asociality and anhedonia) and an expressive deficit dimension (EXP, including blunted affect and alogia).^4,24–26^ This model is consistent with the observation that different behavioural features, neurophysiological bases as well as clinical and social outcomes are associated with the two dimensions.^2,6,27–36^

However, evidence from recent multicentre studies utilising confirmatory factor analysis (CFA) has questioned the adequacy of this two-factor model.^37–43^ These studies suggested that a five-factor model or a hierarchical model better fit the data, irrespective of assessment scale, sample nationality/language or stage of illness.^37,38,40,42,43^ These findings indicate that conceptualising negative symptoms in relation to the MAP and EXP dimensions may not capture the complexity of the construct, and support a more complex view of negative symptoms, aligned with the five NIMH-MATRICS consensus domains.^38,39,44–46^

## Latent structure of negative symptoms

To address the latent structure of negative symptoms has very strong pragmatic implications. For instance, exploratory factor analysis studies supporting the two-factor model have influenced researchers and pharmaceutical companies, resulting in the drafting of clinical and pharmacological research protocols.^30,44,45,47^ However, the two-factor model may prevent the identification of pathophysiological mechanisms or therapeutic effects that are unique to one of the five domains. Further studies are required, as there is some preliminary evidence showing distinct pathophysiological correlates of individual negative symptom domains.^48^

A recent network approach to psychopathology conceptualises disorders as systems of interconnected symptoms.^49,50^ Preliminary studies have used this approach to investigate the structure of negative symptoms across different diagnoses and in terms of treatment response.^51–53^ However, the longitudinal stability of this structure remains largely unexamined, especially with second-generation assessment tools aligned with the current conceptualisation.^54–57^

To address this gap, the primary aim of the present study was to delve deeper into the structure of negative symptoms over time, utilising network analysis. This study seeks to investigate the temporal stability of the negative symptom network over a 4-year period in a representative sample of individuals diagnosed with schizophrenia. By doing so, we aimed to enhance our understanding of the interplay and evolution of negative symptoms, ultimately contributing to the development of more targeted and effective treatment strategies.

## Method

### Participants

This observational prospective study was carried out as part of the Italian Network for Research on Psychoses.^58–61^

Participants in the study were community-dwelling individuals with schizophrenia who had been stabilised on antipsychotic medications for at least 3 months before enrolment and were seen consecutively at the out-patient clinics of 24 Italian university psychiatric clinics and/or mental health departments.

Participants were recruited between 1 March 2012 and 30 September 2013. All patients recruited by participating centres at baseline were asked to participate in the follow-up study carried out 4 years after the baseline assessment.

Inclusion criteria were a diagnosis of schizophrenia according to DSM-IV, confirmed with the Structured Clinical Interview for DSM-IV, Patient Version (SCID-I/P), and an age between 18 and 65 years. Given that the SCID includes both mandatory questions that correspond to DSM-IV operational criteria and a diagnostic algorithm, the diagnosis of schizophrenia is assigned when the following criteria are met: ‘The disturbance is not attributable to the physiological effects of a substance (e.g., a drug of abuse, a medication) or another medical condition’.^62^ In doing so physicians, as good clinical practice requires, are supported in the differential diagnoses by investigations (electrocardiograms, blood and urine samples, computed tomography/magnetic resonance imaging, electroencephalograms) as needed.

Exclusion criteria were: (a) history of head injury with loss of consciousness in the 4 years between baseline and follow-up; (b) progressive cognitive decline possibly caused by dementia or other neurological illness diagnosed in the past 4 years; (c) history of alcohol and/or substance misuse in the past 6 months; (d) current pregnancy or nursing; (e) inability to give informed consent; and (f) treatment modifications and/or hospital admission due to symptom exacerbation in the previous 3 months.

The authors assert that all procedures contributing to this work comply with the ethical standards of the relevant national and institutional committees on human experimentation and with the Helsinki Declaration of 1975, as revised in 2008. All procedures involving human subjects/patients were approved by the Ethics Committee ‘Comitato Etico Università degli Studi della Campania ‘Luigi Vanvitelli’ – Azienda Ospedaliera Universitaria ‘Luigi Vanvitelli’ – AORN ‘Ospedali dei Colli’’ on 9 February 2012 (Protocol number 73, baseline study) and on 9 October 2015 (Protocol number 1382, follow-up study). After receiving a comprehensive explanation of the study procedures and goals, each participant gave written informed consent to participate.

### Clinical assessment

Evaluation of negative symptoms was conducted using the Italian version of the Brief Negative Symptom Scale (BNSS).^22,63^ The scale consists of 13 items organised into six subscales (five negative symptom subscales: anhedonia, asociality, avolition, blunted affect and alogia; and a control subscale: lack of distress). All the items are rated on a 7-point (0–6) scale, thus ranging from absent (0) to moderate (3) to extremely severe (6) symptoms. According to the current conceptualisation of negative symptoms and similar research, BNSS item 4 (‘lack of normal distress’) was left out of the statistical analysis as it is not a negative symptom.^1,40^

The assessment of positive symptoms and disorganisation was conducted using the Positive and Negative Syndrome Scale (PANSS).^64^ In accordance with Wallwork and colleagues^65^ the positive dimension was determined through the sum of the scores on the following PANSS items: delusions (P1), hallucinatory behaviour (P3), grandiosity (P5) and unusual thought (G9). The disorganisation dimension was determined by adding the scores on the following PANSS items: conceptual disorganisation (P2), difficulty in abstract thinking (N5) and poor attention (G11). Depressive symptoms were assessed using the Calgary Depression Scale for Schizophrenia (CDSS).^66^ Last, the St. Hans Rating Scale (SHRS) was used to evaluate extrapyramidal symptoms.^67^ These clinical evaluations were conducted both at baseline and at the 4-year follow-up visit.

### Statistical analysis

Network analysis was carried out on BNSS items at baseline and follow-up. Starting from a network built on partial correlations, where the association between each pair of nodes was controlled for the influence of all the other nodes, an adaptive least absolute shrinkage and selection operator (LASSO) network was obtained by assigning penalties to partial correlations between variables to make small correlations shrink to 0. A tuning parameter of 0.5 was used to control for the sparsity of the network. Because the study variables were not normally distributed, a non-paranormal transformation was applied to the data. The network graphical representation, in which variables are shown as nodes and their correlations are depicted as edges, was based on the Fruchterman–Reingold algorithm, which places strongly associated nodes at the centre of the graph and weakly associated ones at the periphery. To further facilitate readability, only correlations of 0.05 or more were included in the network diagram. The centrality indices of betweenness, closeness and strength were used to quantify the importance of each node in the adaptive LASSO network. The betweenness of a node equals the number of times that it lies on the shortest path length between any two other nodes. Closeness indicates how easy it is to reach all other nodes from the node of interest and is computed as the inverse of the weighted sum of distances of a given node from all other nodes in the network. Nodes with high betweenness are those that facilitate connections in the network, whereas nodes with high closeness affect the other nodes more quickly or are more affected by the other nodes. Last, the node strength is the sum of the correlations of one node to all other nodes. For each index, higher values reflect higher centrality in the network, but high strength may also derive from very strong correlations between peripheral nodes belonging to the same domain. Centrality plots were created to represent these indices. The robustness of the network solution was assessed by estimating the accuracy of edge weights and the stability of centrality indices using bootstrap analysis.^68^

We used R, version 3.3.3 for Windows (R Foundation for Statistical Computing) to perform the network analysis; specifically, the package ‘qgraph’ was used to obtain the network and centrality indices, and ‘bootnet’ to evaluate the network stability. We investigated whether the BNSS network structure differed between baseline and follow-up by means of the network comparison test using the R package ‘NetworkComparisonTest’.^69^

The network structure invariance test investigates differences in the overall structure of the network. The difference between network structures is measured as the deviation in absolute weighted sum scores of the connections.^70^ This permutation-based test randomly reclassifies individuals from the networks repeatedly and then computes the differences between the subnetworks. The resulting distribution under the null hypothesis, assuming that networks are equal, is used to test the observed difference of the subnetworks.^69,71^ We used the option for dependent samples of the network comparison test to test temporal stability. The global strength invariance test was used to investigate whether the overall level of connectivity was equal across networks. When this test was significant, *post hoc* analyses were carried out to determine which specific edges differed between networks using Bonferroni–Holm correction for multiple comparisons. Overall connectivity was computed as the weighted absolute sum of all edges in the network.^72,73^ The significance level of the network comparison tests was set at *P* < 0.05. Community detection analysis was conducted using the function *cluster_spinglass* of the R package ‘igraph’. The spinglass algorithm was chosen because it handles networks with negative weights, which were present in our data. To account for potential variability in the results based on the initial seed, the analysis was performed 10 000 times, thereby allowing calculation of the frequency of different community structures identified at baseline and follow-up. This rigorous approach allowed for a more comprehensive understanding of the stability of the network community structures and provided a robust estimate of the reproducibility of the findings across multiple iterations.

## Results

Out of 921 individuals enrolled at baseline, 618 provided follow-up data and 612 with complete baseline and follow-up BNSS data were included in the analyses (422 men (69%) and 190 women (31%); mean age at follow-up 45.1 years (s.d. = 11.5)). The detail demographic characteristics of the sample and the ongoing treatment are given in Supplementary Table 1, available at https://doi.org/10.1192/bjo.2023.541.

The clinical characteristics are shown in Table 1: 59.5% of participants showed mild to moderate severity of negative symptoms (BNSS total score <36), 77.6% absent to mild positive symptom severity (PANSS positive dimension score <12); 59.2% PANSS disorganisation dimension score <9; 63.5% showed low levels of depression (CDSS total score <4); and 93.3% no or mild Parkinsonism (SHRS Parkinsonism score <1).

**Table 1:** Clinical characteristics of the sample

	Baseline, mean (s.d.)	Follow-up, mean (s.d.)
BNSS total score	33.3 (16.3)	30.5 (16.3)
PANSS positive	9.7 (4.6)	8.4 (4.2))
PANSS disorganisation	8.6 (3.8)	8.1 (3.6)
CDSS	3.9 (4)	3.25 (3.7)
SHRS Parkinsonism	0.87 (0.334)	0.85 (1.2)

BNSS, the Brief Negative Symptom Scale; PANSS, Positive and Negative Syndrome Scale; CDSS, Calgary Depression Scale for Schizophrenia; SHRS, St. Hans Rating Scale for extrapyramidal symptoms.

The means and standard deviations of BNSS items are reported in Table 2.

**Table 2:** Mean and standard deviation of Brief Negative Symptom Scale (BNSS) items at baseline and follow-up^a^

BNSS item	Participants, *n*	Baseline		Follow-up	
		Mean	s.d.	Mean	s.d.
1	612	2.86	1.54	2.54	1.57
2	612	2.96	1.57	2.55	1.56
3	612	2.86	1.58	2.61	1.62
5	612	3.28	1.57	2.88	1.60
6	612	3.01	1.59	2.76	1.58
7	612	2.87	1.61	2.59	1.61
8	612	2.82	1.61	2.59	1.57
9	612	2.71	1.67	2.61	1.59
10	612	2.64	1.77	2.53	1.66
11	612	2.69	1.79	2.54	1.65
12	612	2.21	1.72	2.06	1.69
13	612	2.42	1.79	2.27	1.73

BNSS items: 1, intensity of pleasure during activities; 2, frequency of pleasurable activities; 3, intensity of expected pleasure from future activities; 5, asociality behaviour; 6, asociality internal experience; 7, avolition behaviour; 8, avolition internal experience; 9, facial expression; 10, vocal expression; 11, expressive gestures; 12, quantity of speech; 13, spontaneous elaboration.

a. All item scores decreased significantly (*P* < 0.001) from baseline to follow-up.

**Fig. 1:**
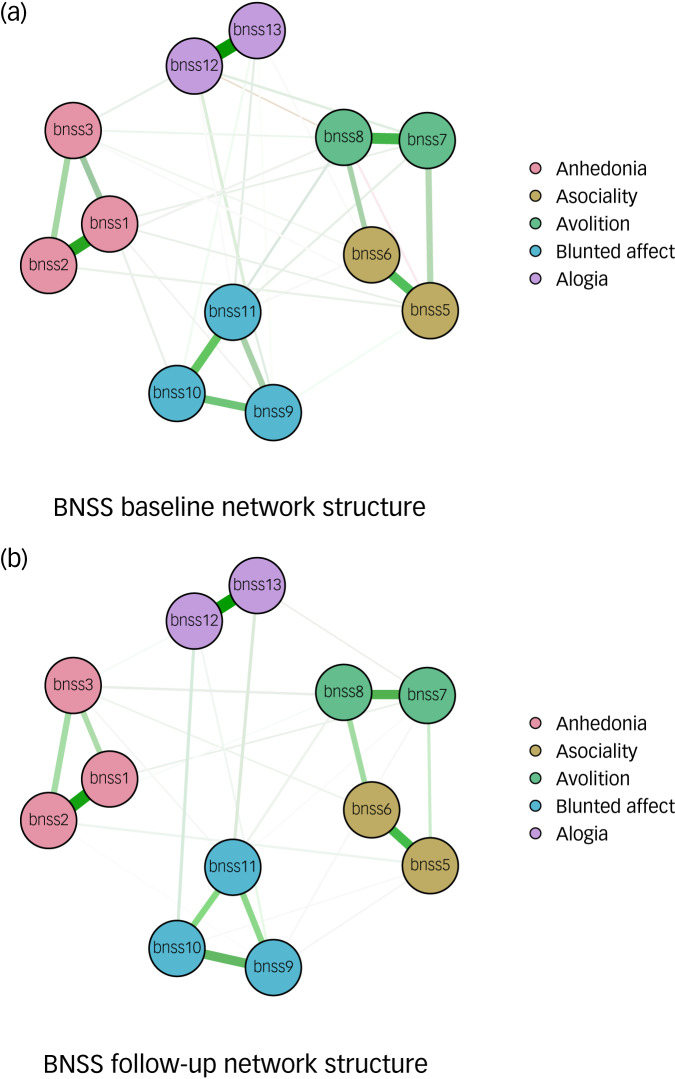
Network structures for Brief Negative Symptom Scale (BNSS) symptoms at (a) baseline and (b) follow-up. Node colours reflect the five domains defined by the NIMH-MATRICS consensus conference and validated by Strauss et al.^40^ BNSS items: 1, intensity of pleasure during activities; 2, frequency of pleasurable activities; 3, intensity of expected pleasure from future activities; 5, asociality behavior; 6, asociality internal experience; 7, avolition behaviour; 8, avolition internal experience; 9, facial expression; 10, vocal expression; 11, expressive gestures; 12, quantity of speech; 13, spontaneous elaboration. The five negative symptom domains identified by the network analysis are: anhedonia, BNSS items 1–3; asociality, 5–6; avolition, 7–8; blunted affect, 9–11; alogia, 12–13. Item 4, measuring the lack of normal distress, was excluded from the analyses, consistent with Strauss et al.^40^

Figure 1 shows the baseline and follow-up networks of BNSS symptoms. Bootstrap analysis revealed that the edge weights were accurate (had small confidence intervals) at baseline and follow-up. In addition, the edge weights were relatively stable until 50% of nodes were removed (Supplementary Figs S1 and S2).

The result of the network invariance test indicated that the network structure was unchanged over time (network invariance test score 0.13, *P* = 0.169), whereas the global strength decreased significantly over time (6.28 at baseline and 5.79 at follow-up; global strength invariance test score 0.48, *P* = 0.016), suggesting that the level of connectivity was reduced at follow-up and at least one edge changed over time. Specifically, we found that six edges changed significantly over time (bnss5–bnss10, *P* = 0.021; bnss7–bnss12, *P* = 0.013; bnss10–bnss12, *P* = 0.004; bnss3–bnss13, *P* = 0.001; bnss6–bnss13, *P* = 0.013; bnss7–bnss13, *P* = 0.003).

The community analysis provided support for four or five domains of the BNSS: anhedonia, asociality, avolition, blunted affect and alogia. Specifically, although anhedonia, blunted affect and alogia communities remained stable at baseline and follow-up, the asociality and avolition items were located in two separate communities in 33.6% of the iterations at baseline and in 63.9% of the iterations at follow-up. Conversely, these items were grouped together into a single domain in 66.1% of the iterations at baseline and 36.1% at follow-up (Supplementary Figs S3 and S4).

## Discussion

The current study used network analysis, a complex and innovative mathematical technique, to investigate the structure of negative symptoms and its stability over time in a sample of people with schizophrenia evaluated at baseline and at 4-year follow-up.

Our findings indicated that the 12 items of the BNSS (with the item ‘lack of normal distress’ being excluded from the analysis, according to the current conceptualisation of negative symptoms and similar research),^2,40^ through strong intra-domain connections, were structured in distinct domains. This network structure was unchanged between baseline and follow-up, whereas its global strength decreased significantly, thus suggesting that the influence of certain items on others diminished over the 4-year period, although the underlying structure of connections remained constant. Furthermore, performing a community analysis, we found that anhedonia, blunted affect and alogia communities remained stable at the two time points, whereas avolition and asociality were located in the same domain at baseline, but they constituted two different domains at follow-up. Therefore, overall, our results indicated a four- or five-factor model of negative symptoms at baseline and a five-factor model at follow-up.

### The five-factor versus the hierarchical model

Our results, despite minor variations, are in line with those presented by Strauss and colleagues.^51^ Although Strauss et al included the BNSS item ‘lack of normal distress’ in their analyses, we chose to exclude this item since it is unclear whether this aspect belongs to the current negative symptom construct or whether it is part of other psychopathological constructs.^2^

Overall, our results partially support the five-domain solution identified in 2005 by the NIMH-MATRICS consensus statement.^51^ These results concur with those of recent confirmatory factor analysis (CFA) studies, despite modest variations in sample sizes and statistical analyses used.^37–43^ These previous investigations showed that the five-factor (five individual negative symptoms: avolition, anhedonia, asociality, blunted affect and alogia) and also the hierarchical model (MAP and EXP as second-order dimensions; five factors as first-order dimensions) offered a great fit, whereas one- and two-factor models performed poorly. Interestingly, the good fit for the hierarchical model should not be interpreted as further support for the two-factor model but rather as a confirmation of the five-factor model, since in the hierarchical model, MAP and EXP are considered second-order dimensions. Therefore, the model of negative symptoms that best accounts for the latent structure of these symptoms is the five-domain one, since all negative symptom ratings in the hierarchical model are directly influenced by primary dimensions.^40^

The results of this network analysis are to a large extent consistent with a four- or five-domain conceptualisation of negative symptoms.

#### The longitudinal network structure stability

The network analysis adds to previous CFA findings on interconnections between negative symptoms. It is of great importance to underline that our negative symptom network structure, in line with previous findings,^51^ indicated not only that items within the five domains cluster together, but also that they have minimal interactions with one another, suggesting a stronger reciprocal influence of the items within each domain and a lower association between items of different domains.

Furthermore, the results of our study indicate that the negative symptom structure derived from a second-generation, culturally unbiased and largely validated instrument such as the BNSS is longitudinally stable.

Despite the latest efforts to describe the latent structure of negative symptoms, much less attention has been focused on invariance of their longitudinal structure. The distinction between stable and unstable symptom clusters may aid in the improvement of diagnostic limits, the prediction of outcome and the identification of specific symptoms that might be prognostically significant. As regards negative symptom stability over time, various studies have investigated their long-term course, reporting controversial findings (e.g. relative stability over time but also reversibility or fluctuation in symptoms over time).^74–77^ However, these studies used the PANSS or the Scale for the Assessment of Negative Symptoms (SANS) and, although they investigated the course of negative symptoms over time, they did not evaluate the stability of the negative symptom structure. To our knowledge, only two studies were carried out with the aim of investigating negative symptom structure stability over time, using a network analysis^55^ or a CFA.^56^ In the study of Levine & Leucht,^55^ negative symptoms were assessed using the SANS in people with chronic schizophrenia and predominant negative symptoms. The authors used a network analysis and found preliminary evidence for a negative symptom severity network consisting of four dimensions (affect, poor responsiveness, lack of interest and apathy/inattentiveness), with these results being replicable at baseline and follow-up (60 days).^55^ These results are heavily influenced by the inadequate assessment instrument, which includes cognitive deficits among negative symptoms, does not distinguish anhedonia and asociality and is based only on behaviour, with poor evaluation of anhedonia and avolition. An antipsychotic-naive first-episode schizophrenia sample was used in the study by Kagan and colleagues to explore the longitudinal invariance of the negative symptom dimension using CFA on PANSS scores at baseline and at 10-week follow-up.^56^ In their study design, the authors examined the longitudinal invariance of the unidimensional and bidimensional models of negative symptoms and found that the unidimensional one had a good fit at baseline and acceptable fit at 10-week follow-up.^56^ However, comparisons between these two studies and our research are not possible in terms of methodology,^56^ assessment instruments used^55,56^ and population included (individuals with chronic schizophrenia and individuals with first-episode psychosis).^56^

### Implications

Overall, the findings of the present study could have implications for clinical practice. First, considering the results of the previous exploratory factor analyses, DSM-5 based the description of negative symptoms on the two dimensions ‘MAP’ and ‘EXP’, with consequent risk of inaccurate diagnoses that do not capture the complexity of the construct of negative symptoms.^27^ Considering current findings, future versions of the DSM might take each of the five domains into account separately. Second, the analysis of the nodes’ centrality and the density of intra- and interdomain relationships may provide valuable information from a therapeutic perspective. An effective treatment targeting densely connected networks could in fact be more effective in inducing a global improvement in negative symptoms, compared with an effective treatment targeting weakly connected domains.^40^ The findings of the study by Strauss and colleagues^53^ demonstrated that roluperidone^78^ improved negative symptoms by reducing the level of centrality of avolition, thus supporting the idea that a global improvement of negative symptoms requires decoupling the influence of motivational processes from other domains of negative symptoms.^53^ Last, correct characterisation of the negative symptom structure and its longitudinal evaluation can allow the identification of pathophysiological mechanisms of the different domains and improving the design of pharmacological/rehabilitative treatment trials, which would be precluded from studying the correlates of the two factors on which the attention of research has been concentrated in recent years.

Our study emphasises the multidimensional nature of negative symptoms. This research underscores the need for continued exploration in this area, to refine psychopathological classifications and develop effective treatment strategies for schizophrenia.

### Limitations

Certain limitations of this study should be taken into account. For instance, participants were predominantly male, which may limit the generalisability of our results. However, we have to note that a higher severity of negative symptoms has been previously reported in males with schizophrenia compared with females.^79^ In addition, positive symptoms, extrapyramidal side-effects and depression are possible sources of secondary negative symptoms, as reported in the introductory section. Therefore, these factors might account for some influences on the presented results. However, our sample comprised clinically stable individuals with schizophrenia, with absent to mild positive and disorganisation symptom severity (PANSS positive dimension mean score <12; PANSS disorganisation dimension mean score <9), low mean level of depression (CDSS total score <4) and Parkinsonism (SHRS Parkinsonism score <1), far below the threshold of clinical significance, thus limiting possible sources of secondary negative symptoms.

## Supporting information

Rucci et al. supplementary material Rucci et al. supplementary material

## Data Availability

The data that support the findings of this study are available from the corresponding author, G.M.G., on reasonable request.
