# Supplementary material for: The structure stability of negative symptoms: longitudinal network analysis of the Brief Negative Symptom Scale in people with schizophrenia
Source: BJPsych Open. 2023 Sep 7;9(5):e168. doi: 10.1192/bjo.2023.541 (PMC10594087; doi:10.1192/bjo.2023.541)
Supplement: Rucci et al. supplementary material [file S2056472423005410sup001.docx]

The Structure Stability of Negative Symptoms: Longitudinal Network Analysis of the Brief Negative Symptom Scale in Subjects with Schizophrenia

Paola Rucci ^1,†^, Edoardo Caporusso ^2,†^, Francesco Sanmarchi ^1^, Giulia M. Giordano ^2,^*, Armida Mucci ^2^, Luigi Giuliani ^2^, Pasquale Pezzella ^2^, Andrea Perrottelli ^2^, Paola Bucci ^2^, Paola Rocca ^3^, Alessandro Rossi ^4^, Alessandro Bertolino ^5^, Silvana Galderisi ^2^, Mario Maj ^2^ and Italian Network for Research on Psychoses ^‡^

^1^ Department of Biomedical and Neuromotor Sciences, University of Bologna, Bologna, Italy,

^2^ Department of Psychiatry, University of Campania “Luigi Vanvitelli”, Naples, Italy

^3^ Department of Neuroscience, Section of Psychiatry, University of Turin, Turin, Italy

^4^ Section of Psychiatry, Department of Biotechnological and Applied Clinical Sciences, University of L'Aquila, L'Aquila, Italy

^5^ Department of Basic Medical Science, Neuroscience and Sense Organs, University of Bari 'Aldo Moro', Bari, Italy

***** Correspondence: [giuliamgiordano@gmail.com](mailto:giuliamgiordano@gmail.com); Tel.: 0815666512

**Index**

**Table S1: Detailed Characteristics of study participants at follow-up (N=612).**

**Figure S.1. Bootstrapped confidence intervals of estimated edge weights for the regularized network of BNSS symptoms at baseline and follow-up. The red line indicates the sample values and the grey area the 95% CIs.**

**Figure S.2. Average correlations between edge weights vs. percentage of sampled nodes.**

**Figure S.3. Community analysis of the BNSS follow-up network structure (5 communities).**

**Figure S.4. Community analysis of the BNSS follow-up network structure (4 communities).**

**Table S.1. Detailed characteristics of study participants at follow-up (N=612).**

| **Variables** | **Percentage frequency or mean±SD** |
| --- | --- |
| **Gender (% males)** | 69.0 |
| **Age (years, mean±SD)** | 45.1±10.5 |
| **Married (%)** | 7.5 |
| **Working (%)** | 34.4 |
| **Education (years, mean±SD)** | 11.7±3.4 |
| **Stable affective relationships (%)** | 18.9 |
| **Legal problems (%)** | 1.3 |
|  |  |
| **Current drug treatment (%)** | 99.2 |
| *antipsychotics (%)* | 98.8 |
| *first generation* | 13.1 |
| *second generation* | 73.6 |
| *first and second generation* | 13.3 |
| *antidepressants (%)* | 17.6 |
| *mood stabilizers (%)* | 26.0 |
| *anxiolytics (%)* | 32.8 |
| *anticholinergics (%)* | 9.5 |
|  |  |
| **Polypharmacy (%)** | 53.8 |
|  |  |
| **Any psychosocial interventions (%)** | 34.3 |
|  |  |
| **Psychotherapy (%)** | 14.9 |
|  |  |
| **Substance abuse (%)** | 4.9 |
| **Alcohol abuse (%)** | 4.6 |
| **Smoking (%)** | 42.0 |
| **Suicide attempts (%)** | 3.4 |

*Number of patients with missing data: Working 8; Stable affective relationships 5; Legal problems 3; Current drug treatment 2; Antipsychotics 9; antidepressants 9; mood stabilizers 9; anxiolytics 9; anticholinergics 9; Polypharmacy 9; Any psychosocial interventions 3; Psychotherapy 6; Substance abuse 1; Alcohol abuse 1; Smoking 9.*

**Bootstrap analysis**

The accuracy of edge weights was measured by the 95% confidence intervals (CIs) obtained from 1000 bootstrap samples drawn from the study population at baseline and follow-up: the narrower the CI, the more accurate is the estimate of the edge weight. Figure S1 (grey areas) shows that the CIs of edge weights estimates are narrow and that edges with the highest absolute partial correlations are significantly different from most of the other edges (CIs do not overlap). The edge-weights estimations are therefore accurate.

The stability of edge weights was measured by correlating the edge weights in the original sample with those obtained by sampling increasing lower percentages of nodes. Figure S2 shows that the correlations remain high (>0.90) until less than 50% of nodes are sampled.

**Figure S.1.** Bootstrapped confidence intervals of estimated edge weights for the regularized network of BNSS symptoms at baseline and follow-up. The red line indicates the sample values and the grey area the 95% CIs.

**Baseline**


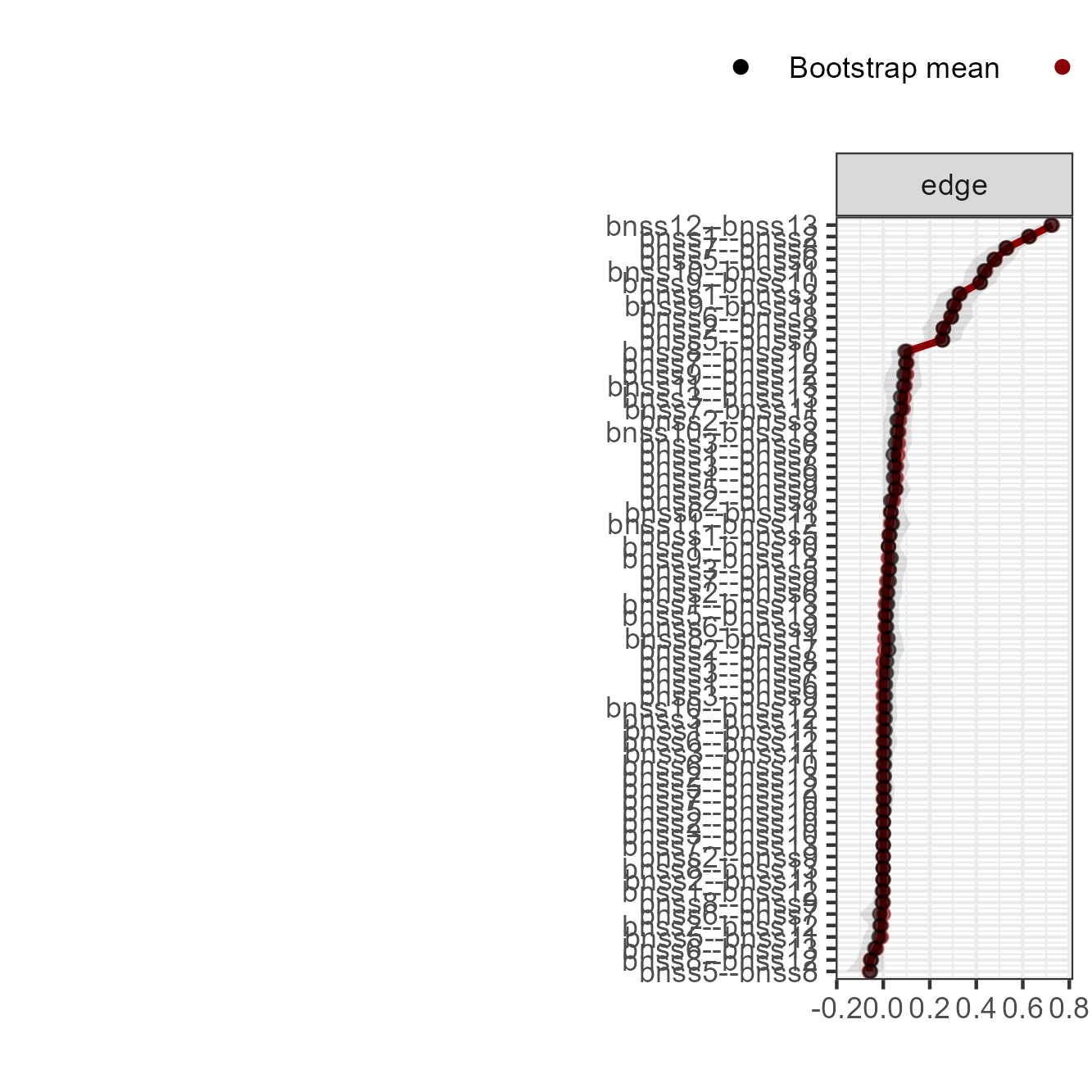


**Follow-up**


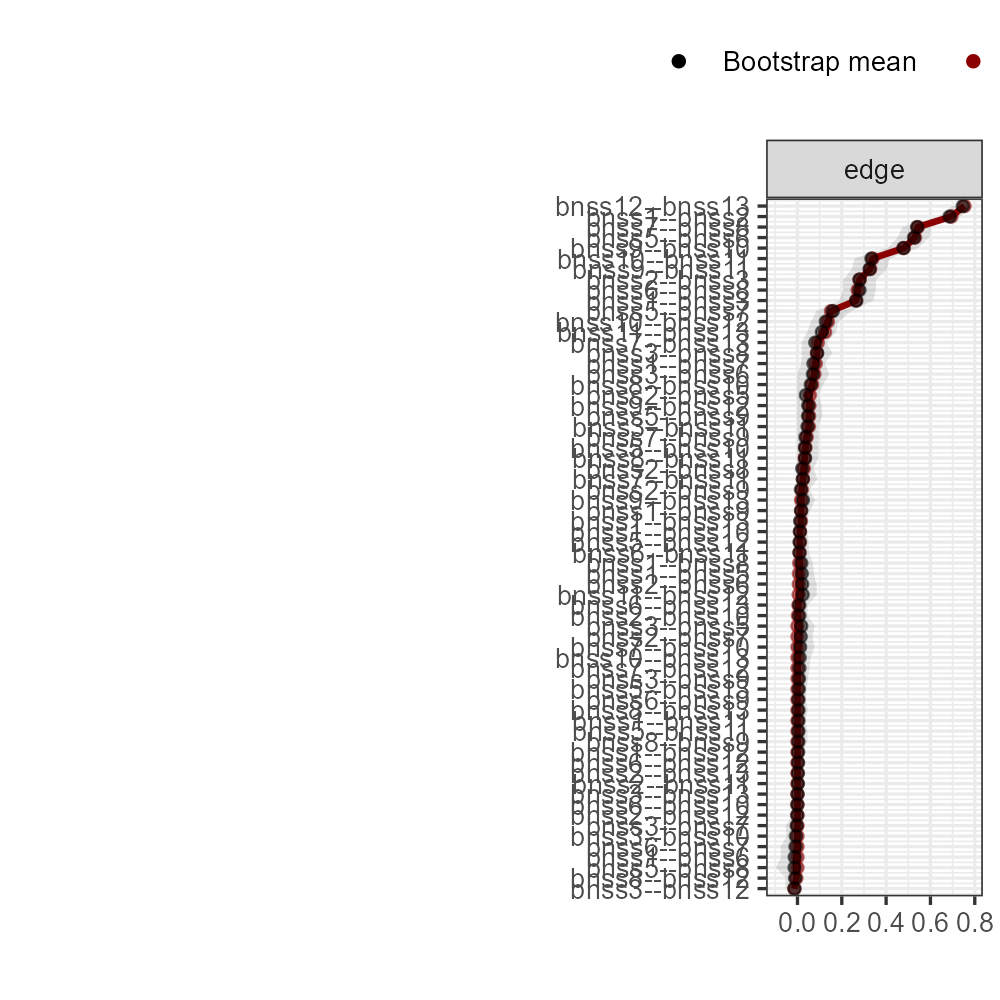


**Figure S.2.** Average correlations between edge weights vs. percentage of sampled nodes.

**Baseline**


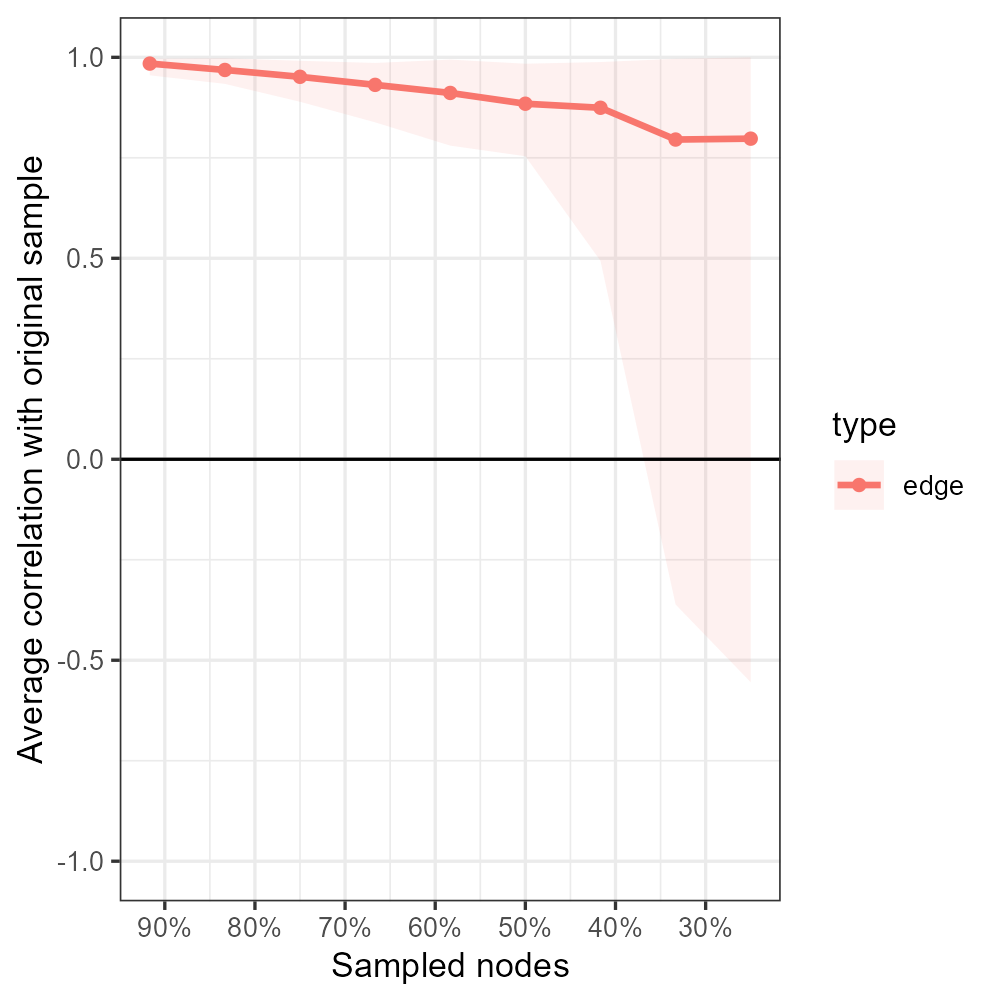


**Follow-up**


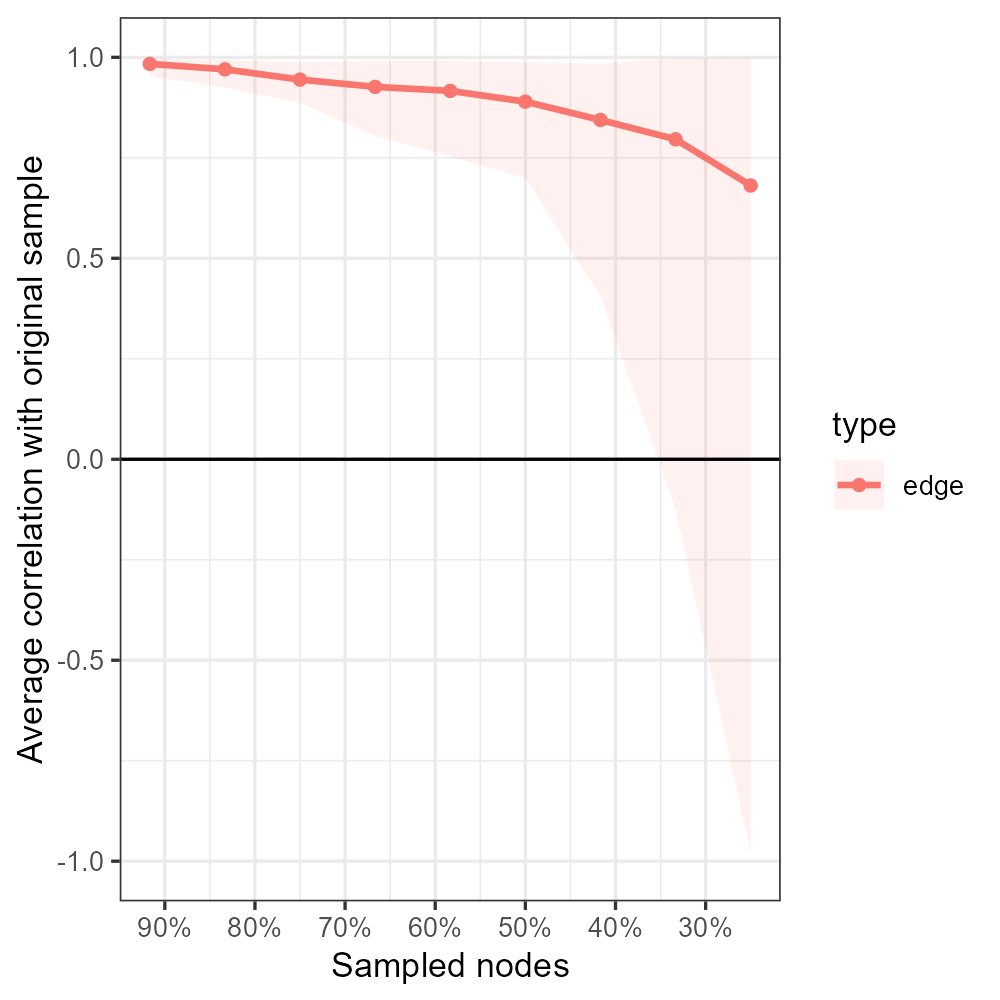


**Figure S.3.** Community analysis of the BNSS follow-up network structure (5 communities).

**
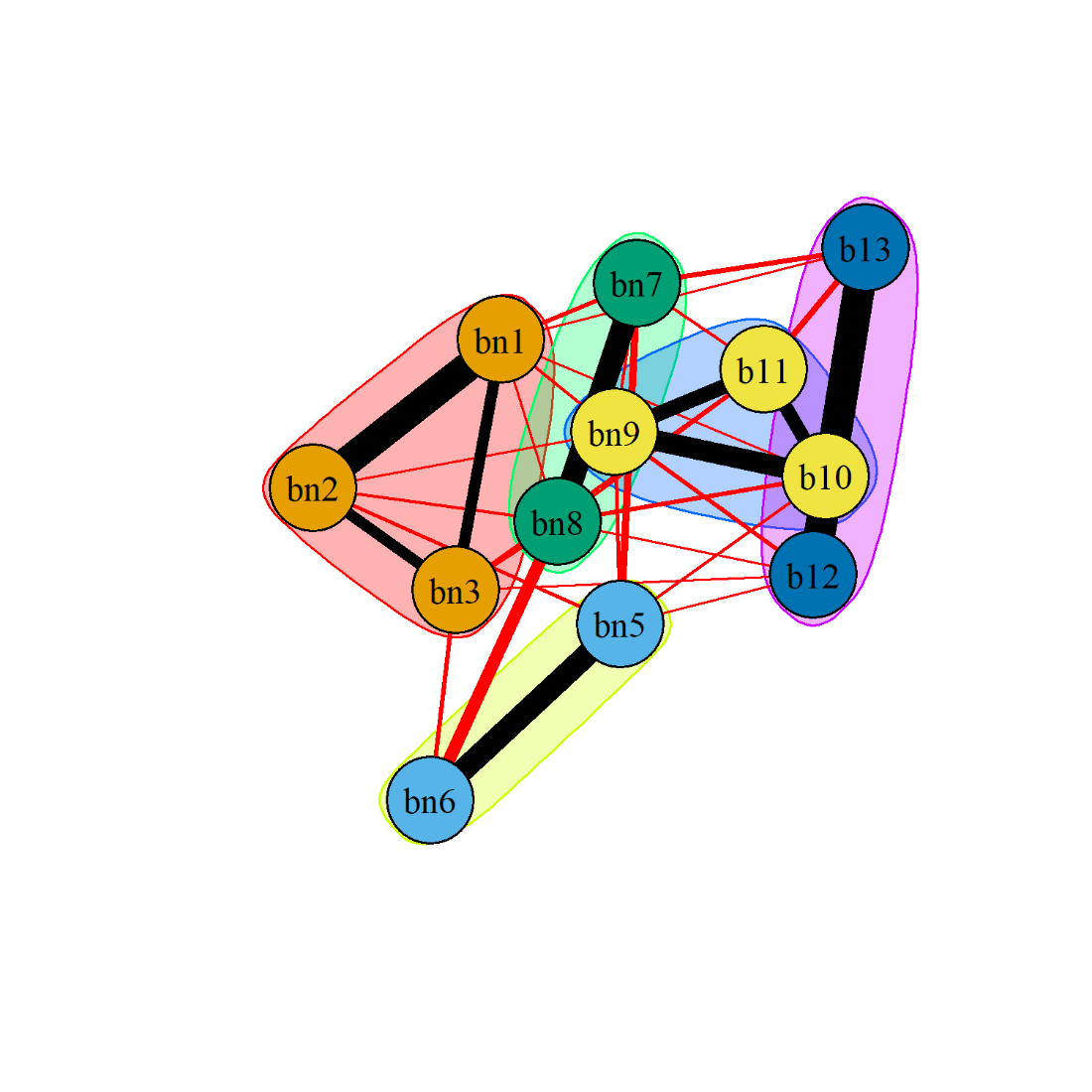
**

**Figure S.4.** Community analysis of the BNSS follow-up network structure (4 communities).

**
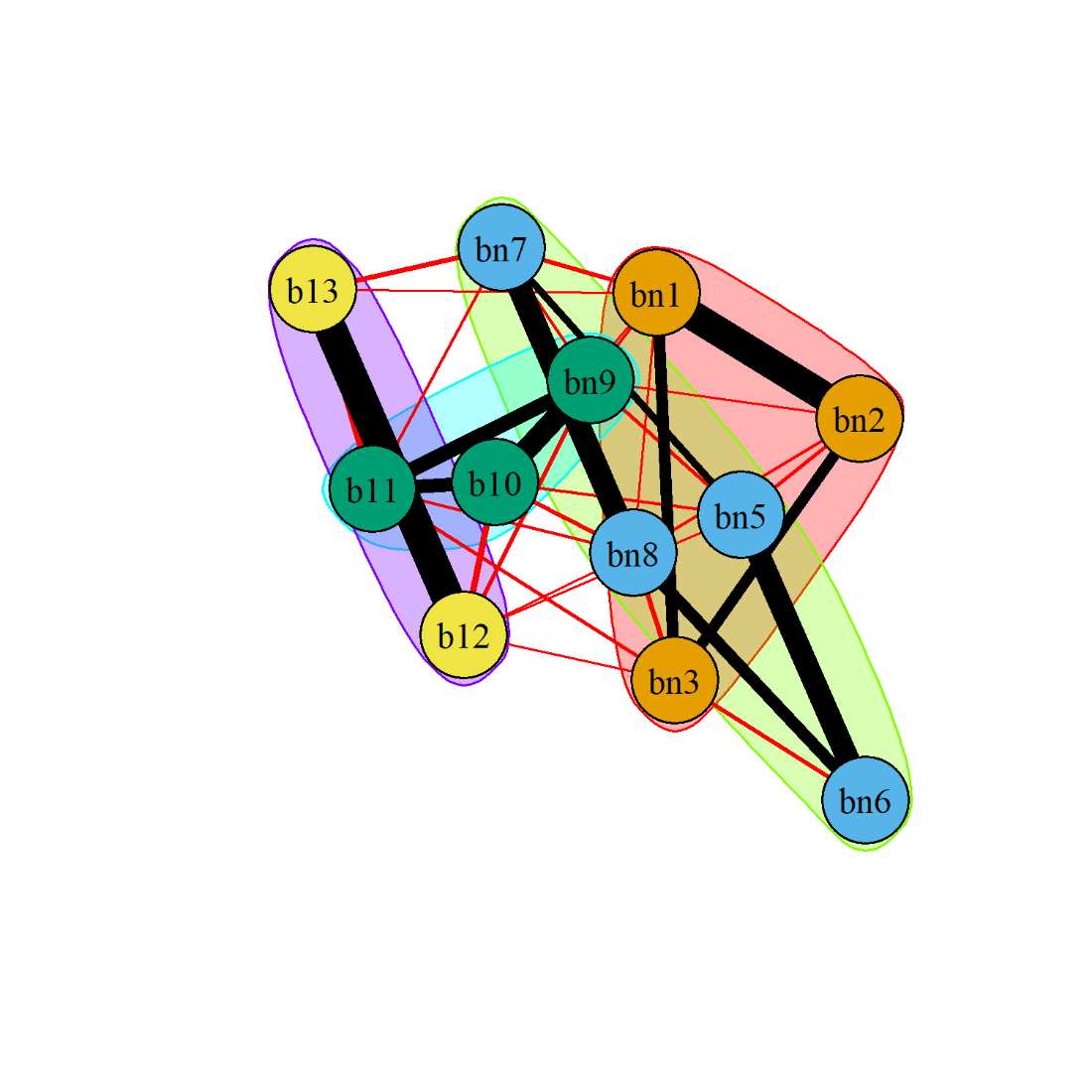
**
